# Determination of birth-weight centile thresholds associated with adverse perinatal outcomes using population, customised, and Intergrowth charts: A Swedish population-based cohort study

**DOI:** 10.1371/journal.pmed.1002902

**Published:** 2019-09-20

**Authors:** Matias C. Vieira, Sophie Relph, Martina Persson, Paul T. Seed, Dharmintra Pasupathy

**Affiliations:** 1 Department of Women and Children’s Health, School of Life Course Sciences, Faculty of Life Sciences and Medicine, King’s College London, London, United Kingdom; 2 Department of Obstetrics and Gynaecology, School of Medicine, Pontifícia Universidade Católica do Rio Grande do Sul, Porto Alegre, Brazil; 3 Department of Medicine, Solna, Clinical Epidemiology Unit, Karolinska Institutet, Stockholm, Sweden; Cambridge University, UNITED KINGDOM

## Abstract

**Background:**

Although many studies have compared birth-weight charts to determine which better identify infants at risk of adverse perinatal outcomes, less attention has been given to the threshold used to define small or large for gestational age (SGA or LGA) infants. Our aim was to explore different thresholds associated with increased risk of adverse perinatal outcomes using population, customised, and Intergrowth centile charts.

**Methods and findings:**

This is a population-based cohort study (Swedish Medical Birth Registry), which included term singleton births between 2006 and 2015 from women with available data on first-trimester screening. Population, customised, and Intergrowth charts were studied. Outcomes included cesarean section, postpartum haemorrhage, severe perineal tear, Apgar score at 5 minutes, neonatal morbidity, and perinatal mortality. Odds for each outcome were assessed in intervals of 5 centiles of birth weight (reference being 40th–60th centiles) using logistic regression. Intervals of 5% of the population were also explored. Sensitivity for fixed false-positive rates (FPRs) was reported for neonatal outcomes. Data from 212,101 births were analysed. Mean age was 33 ± 5 years, 48% of women were nulliparous, and 80% were born in Sweden. Prevalence of SGA (<10th centile) was 10.1%, 10.0%, and 3.1%, and prevalence of LGA (>90th centile) was 10.0%, 8.2%, and 25.1%, assessed using population, customised, and Intergrowth charts, respectively. In small infants, the risk of perinatal mortality was consistently increased below the 15th, 10th, and 35th birth-weight centiles for the respective charts (odds ratio [OR] 1.59, 95% confidence interval [CI] 1.05–2.39, *p* = 0.03 for 10th–15th population centile; OR 2.54, 95% CI 1.74–3.71, *p* < 0.001 for 5th–10th customised centile; OR 1.81, 95% CI 1.07–3.04, *p* = 0.03 for 30th–35th Intergrowth centile). The strength of association with adverse perinatal outcomes was different between infants below the 5th birth-weight centile for each chart (OR 4.47, 95% CI 3.30–6.04, *p* < 0.001 for the population chart; OR 5.78, 95% CI 4.22–7.91, *p* < 0.001 for the customised chart; OR 10.74, 95% CI 7.32–15.77, *p* < 0.001 for the Intergrowth chart) but similar in the smallest 5% of the population (OR 4.34, 95% CI 3.22–5.86, *p* < 0.001 for the population chart; OR 5.23, 95% CI 3.85–7.11, *p* < 0.001 for the customised chart; OR 4.69, 95% CI 3.47–6.34, *p* < 0.001 for the Intergrowth chart). For a fixed FPR of 10%, different thresholds for each chart achieved similar sensitivity for perinatal mortality in small infants (29% for all charts). Similar behaviour of different thresholds and similar risk/sensitivity for fixed FPR were observed in relation to other outcomes and for LGA infants. Limitations of this study include the relative homogeneity of the Swedish population, which limits generalisability to other populations; customised centiles may perform differently in populations with increased heterogeneity of ethnic background.

**Conclusions:**

The risk of adverse outcomes was consistent across proportions of the population but did not reflect fixed thresholds, such as the 10th or 90th centiles, across different growth charts. Chart-specific thresholds for the population should be considered in clinical practice.

## Abstract 2

Matias C Vieira and colleagues reveal the associations of perinatal outcomes in infants when using growth charts in the 10th and 90th centiles.

## Author summary

**Why was this study done?:**

- Different growth charts are currently used to identify babies that have suboptimal or excessive growth. There is considerable debate on which of these charts should be used.
- Most studies comparing different growth charts have not explored the impact of using different thresholds to define small or large for gestational age infants, other than the 10th and the 90th centiles.

**What did the researchers do and find?:**

- In this study, we explored different thresholds associated with increased risk of adverse perinatal outcomes using population, customised, and Intergrowth charts.
- Increased risk of adverse outcomes in small and large infants occurred at different thresholds for each chart and were also different according to the outcome studied.
- The strength of association with adverse perinatal outcomes was different between infants below the 5th birth-weight centile for each chart (odds ratio [OR] 4.47, 95% confidence interval [CI] 3.30–6.04, *p* < 0.001 for the population chart; OR 5.78, 95% CI 4.22–7.91, *p* < 0.001 for the customised chart; OR 10.74, 95% CI 7.32–15.77, *p* < 0.001 for the Intergrowth chart) but similar in the smallest 5% of the population (OR 4.34, 95% CI 3.22–5.86, *p* < 0.001 for the population chart; OR 5.23, 95% CI 3.85–7.11, *p* < 0.001 for the customised chart; OR 4.69, 95% CI 3.47–6.34, *p* < 0.001 for the Intergrowth chart).
- The performance of these three charts to detect infants with adverse outcomes, such as perinatal mortality, was similar when thresholds that reflect a false-positive rate of 10% were used for each chart.

**What do these findings mean?:**

- Our findings suggest that either of the three charts in this study could be used in clinical practice, each having a similar ability to identify babies with adverse outcomes if thresholds that are specific to each individual chart are used.
- Further studies should explore whether this approach is also applicable to other populations. Researchers and clinicians should strive to achieve consensus in developing chart-specific thresholds.

## Introduction

Infants with abnormal fetal growth have an increased risk of adverse perinatal outcomes [1–3]. Both small for gestational age (SGA) and large for gestational age (LGA) infants face excess risk of perinatal mortality and morbidity [3–5]; they are also at increased risk of long-term consequences including childhood obesity and metabolic disease later in life [6,7].

Growth charts, with varying methodologies, are used to identify SGA (usually defined as birth weight < 10th centile) and LGA infants (usually defined as birth weight > 90th centile). Population charts describe birth-weight distributions adjusted for gestational age, with or without adjustment for fetal sex. Customised birth-weight centiles demonstrate a more personalised assessment of growth potential by additionally accounting for maternal characteristics associated with fetal growth. These include maternal ethnicity, height, weight, and parity [8]. It has been reported that customised centiles identify an additional group of infants at risk of adverse perinatal outcomes compared with population centiles [2,9]. Some authors have suggested this association is largely due to customised centiles increasing the incidence of SGA identified in the preterm period [10,11]. The recent publication of two new international standards, the Intergrowth 21st Project and WHO Fetal Growth Charts, which adjust for fetal sex and gestational age, are based on the principle that infants worldwide should have the same growth potential. Given their recent release, there is lack of complete understanding of the performance of these charts. Some studies comparing these charts have suggested that international charts may not accurately identify infants at risk of adverse outcomes related to abnormal growth [12–14].

Although many of the previous studies have aimed to determine which charts better identify infants at risk of adverse perinatal outcomes, less attention has been given to the threshold used to define SGA and LGA infants [15]. Notably, there is a paucity of data about both neonatal and maternal morbidity outcomes such as cesarean section, postpartum haemorrhage, severe perineal trauma, Apgar score, and neonatal morbidity. Recognising the optimal thresholds for assessing the risk of morbidity for each growth chart will ensure that they are used appropriately in a population-specific clinical setting. The aim of this study was to explore different thresholds associated with increased risk of adverse perinatal outcomes using population, customised, and Intergrowth centile charts.

## Methods

### Study population

This is a population-based cohort study using data from birth records in the Swedish Medical Birth Registry (SMBR). Woman residents in Sweden with singleton births between 2006 and 2015, who also had first-trimester screening data available in the Swedish Pregnancy Registry, were included. Participation in public prenatal care in Sweden is almost 100%, which means that antenatal and delivery data in SMBR are available for virtually all pregnancies in the country. Records with missing data on gestational age at delivery or infants’ birth weight, women with preterm (birth before 37 weeks’ gestation) or postterm birth (defined here as birth at or after 43 weeks’ gestation), multiple pregnancies, and pregnancies with fetal malformations were excluded. This study was approved by the Regional Ethics Committee in Stockholm, Sweden (2017/1031-32). Individual informed consent was not obtained given the nature of this registry study.

### Outcomes

Maternal outcomes included all cesarean section, emergency cesarean section, postpartum haemorrhage, and severe perineal trauma (third/fourth-degree tear). These outcomes were available directly from the SMBR dataset, except for postpartum haemorrhage, which was identified using codes from the *International Classification of Diseases*, *10th version* (ICD-10; O72 was used to identify postpartum haemorrhage). Neonatal adverse outcomes included Apgar score at 5 minutes, a composite outcome of neonatal morbidity, and perinatal mortality (stillbirth or early neonatal death). Neonatal morbidity was defined as one or more of the following neonatal complications: seizure, pulmonary hypertension, bronchopulmonary dysplasia, hypoxic ischemic encephalopathy, intraventricular haemorrhage grade III or IV, other intracranial haemorrhage, necrotizing enterocolitis, bone fracture, brachial plexus injury, facial palsy, sepsis, and/or cardiac arrest and identified using codes from the ICD-10 (please see S1 Table).

### Birth-weight charts

Exposures of interest were birth-weight centiles measured using three different charts (population, customised, and Intergrowth). Population centiles were internally calculated using birth weight, adjusted for gestational age and infant sex. Customised centiles were calculated using birth weight, adjusted for maternal height, weight, ethnicity and parity, infant sex, and gestation at delivery through the GROW calculator version 8.0.1 [8]. Intergrowth centiles for the study population were defined as birth weight adjusted for gestational age and sex according to the Intergrowth 21st International Standard and were calculated using the International Standards for Size at Birth software version 1.0.6257.25111 [16]. Customised and Intergrowth standards were originally developed using infants from healthy women. A summary of the characteristics of each growth chart explored in this study is provided in Table 1.

**Table 1:** Summary of characteristics of growth charts explored.

Characteristics	Population	Customised	Intergrowth
Population used for development	Swedish population (internally developed using Swedish Medical Birth Registry population available to this study, *n* = 233,379)	Combination of registries and datasets for multiple countries (www.gestation.net)	Pooled data from single-centre cohorts in eight countries (*n* = 20,486)
Estimated fetal weight versus birth weight	Birth-weight chart	Term optimal weight was developed using birth weight. In the preterm period, fetal weight has been estimated using a proportionality formula based on the Hadlock’s fetal weight equation	Birth-weight chart
Descriptive versus prescriptive^a^	Retrospective development of a descriptive chart	Retrospective development of a prescriptive chart	Prospective development of a prescriptive chart
Adjustment/covariates	Gestational age and infant sex	Maternal ethnicity, height and weight, parity, gestational age, and infant sex	Gestational age and infant sex
Underlying principle	Infants in a given population should have a similar growth potential	Infant growth potential is physiologically influenced by maternal and fetal factors	Infants worldwide should have a similar growth potential

^a^Descriptive charts are also known as ‘references’ and describe the estimated fetal weight or birth weight in the whole sample, including women with comorbidities such as preeclampsia or gestational diabetes. Prescriptive charts are also known as standards and aim to report growth in presumably healthy individuals, usually excluding the effect of comorbidities, smoking, and socioeconomic status (amongst other factors). The factors used to presume a healthy population may vary between charts.

### Patient involvement

There was no patient and public involvement in setting the research question or the outcome measures, nor were they involved in developing plans for the study or in interpreting or writing up the results. The full study and its results are published in this open-access journal and are therefore available to participants.

### Statistical analysis

This analysis was performed using all data available, following application of the inclusion and exclusion criteria above. For women with missing height or weight, the bulk customised centile calculator uses the median value of height and weight for the woman’s ethnicity to calculate the customised centile. Country of birth was used as a proxy for ethnicity because this was the only information available. Country of birth was matched to the most suitable country/area in the customised calculator. Population centiles were calculated using the ‘centile’ Stata command [17] and adjusted for gestation at birth in days and fetal sex. Customised and population centiles are routinely calculated to one decimal point, whereas the Intergrowth calculator provides centiles with two decimal points. The following analysis was then performed (S1 Text).

First, for each chart, women were divided into 20 groups based on their infant’s birth-weight centile. Each group represented an interval of 5 birth-weight centiles. Logistic regression models were used to assess associations with adverse outcomes; rate of outcomes for each group were plotted, and the odds ratios (ORs) with 95% confidence intervals (CIs) were calculated. The reference group was defined a priori as infants between the 40th and the 60th centiles for each centile chart. This analysis provided information on the association between the birth-weight centile and adverse outcomes for each chart. A sensitivity analysis using ordinal logistic regression to explore Apgar as continuous was performed to confirm that categorisation of Apgar into binary (<7 at 5 minutes) did not affect the results.

Second, women were divided into 20 equally sized groups ranked by birth-weight centile for each chart. Each group represents 5% of the population. The analysis of associations with adverse outcomes was then repeated using these groups. The reference group included the four groups in the middle of the distribution (i.e., middle 20% of the population). This analysis provided insight on the risk of adverse outcomes specific to the distribution of the study population. These thresholds, used to create 20 equally sized groups, will be referred to as chart-specific thresholds for this population and are reported in Table 2.

**Table 2:** Description of chart-specific thresholds for this study population that produce equally sized groups (with 5% of the population).

Groups^a^		Population centiles	Customised centiles	Intergrowth centiles
Smallest infants	0.0%–4.9% of population	<5	<5.2	<14.8
	5.0%–9.9% of population	<9.9	<10.0	<25.4
	10.0%–14.9% of population	<14.9	<14.5	<34.1
	15.0%–19.9% of population	<19.8	<18.9	<41.7
	20.0%–24.9% of population	<24.8	<23.3	<48.4
	25.0%–29.9% of population	<29.8	<27.6	<54.3
	30.0%–34.9% of population	<34.8	<31.9	<59.8
	35.0%–39.9% of population	<39.8	<36.4	<64.7
	40.0%–44.9% of population	<44.8	<40.8	<69.1
	45.0%–49.9% of population	<49.8	<45.3	<73.3
	50.0%–54.9% of population	≥49.8	≥45.3	≥73.3
	55.0%–59.9% of population	>54.7	>49.9	>77.2
Largest infants	60.1%–65.0% of population	>59.7	>54.7	>80.8
	65.1%–70.0% of population	>64.7	>59.7	>84.1
	70.1%–75.0% of population	>69.7	>64.8	>87.2
	75.1%–80.0% of population	>74.8	>70.1	>90.0
	80.1%–85.0% of population	>79.8	>75.7	>92.6
	85.1%–90.0% of population	>84.8	>81.4	>94.9
	90.1%–95.0% of population	>89.8	>87.6	>97.0
	95.1%–100.0% of population	>94.9	>94.0	>98.7

^a^Each group represents 5% of the population (approximately 10,600 women); groups are ranked by birth-weight centile for each chart. Infants in each group (e.g., 0.0%–4.9% of the population) overlap considerably between charts, but they are not necessarily the same group of infants.

Third, assuming that fetal weight charts reflect birth-weight charts at term [18], diagnostic test performance of different thresholds in each chart was explored in relation to neonatal morbidity and perinatal mortality. For each chart, five thresholds were used for LGA (>95th, >90th, >85th, >80th, and >75th centile) and SGA (<5th, <10th, <15th, <20th, and <25th centile). In addition, thresholds that determine fixed false-positive rates (FPRs) of 5%, 10%, 15%, 20%, and 25% were also explored for each chart. Number and rate of events, sensitivity, and FPR were described for these thresholds.

All statistical analyses were performed using Stata software, version 15.1 (StataCorp, College Station, Texas, United States). This study has been reported in line with STROBE recommendations (S1 STROBE Checklist) [19].

## Results

**Fig 1:**
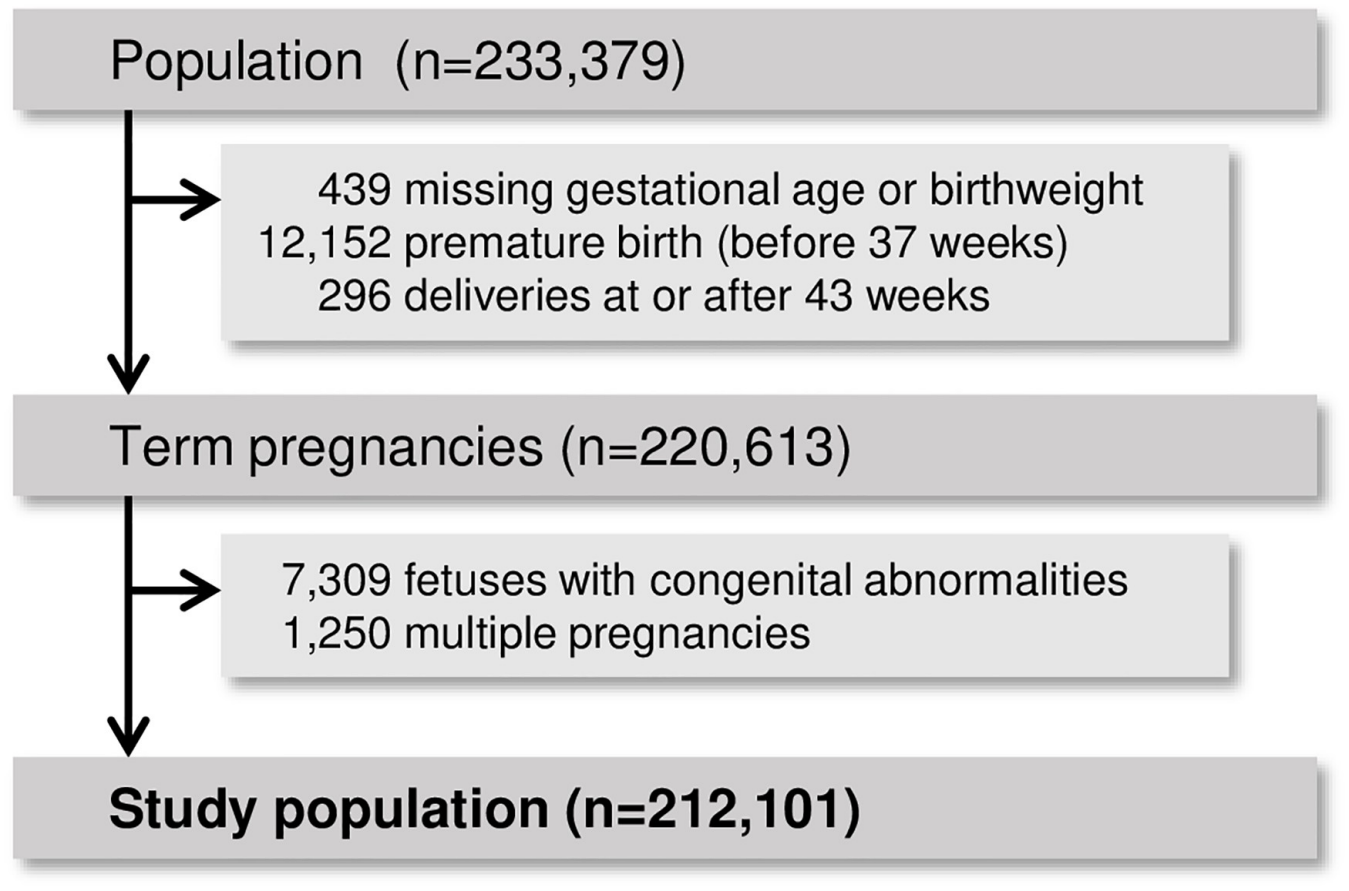
Study population (exclusion criteria are not mutually exclusive).

Amongst 233,379 women that met the inclusion criteria, 212,101 (90.9%) were part of our study population (Fig 1). The mean age of this cohort was 33 ± 5 years, 48% of women were nulliparous, and 80% were born in Sweden. The mean birth weight was 3,594 ± 478 g. Detailed demographic characteristics and pregnancy outcomes in the study population are provided in Table 3. A breakdown of neonatal morbidity composite is provided in S1 Table. Fracture and brachial plexus injury accounted for more than one-third of neonatal morbidities.

**Table 3:** Demographic characteristics and pregnancy outcomes of the study population.

Characteristics/Outcomes	Study population (*n* = 212,101)
	Mean (SD) or frequency (%)
Age (years)	33.1 (4.8)
Parity	
0	101,359 (47.8)
1	75,513 (35.6)
2	26,382 (12.4)
≥3	8,847 (4.2)
Country/region of birth	
Sweden	168,797 (79.6)
The rest of Europe	18,021 (8.5)
Asia	16,706 (7.9)
Africa	3,861 (1.8)
South America	3,010 (1.4)
Other	1,706 (0.8)
Height (cm)^a^	167 (6)
Weight (kg)^a^	67 (12)
BMI category^a^	
Underweight (<18.5 kg/m^2^)	4,723 (2.4)
Normal weight (18.5–24.9 kg/m^2^)	131,273 (66.5)
Overweight (25.0–29.9 kg/m^2^)	43,617 (22.1)
Obesity (≥30.0 kg/m^2^)	17,785 (9.0)
Smoking at booking^a^	7,131 (3.5)
Maternal preexisting disease	
Chronic kidney disease	894 (0.4)
Diabetes mellitus	1,246 (0.6)
Hypertension	1,185 (0.6)
**Pregnancy outcomes**	
Gestation at delivery (weeks)	40.1 (1.2)
All cesarean section	40,382 (19.0)
Emergency cesarean section^b^	19,076 (10.0)
Postpartum haemorrhage	12,882 (6.1)
Severe perineal tear^c^	4,301 (2.0)
Birth weight (grams)	3,594 (478)
Apgar < 7 at 5 minutes^a^	1,935 (0.9)
Neonatal morbidity^d^	2,354 (1.1)
Perinatal mortality	458 (0.2)

^a^Missing value for height (*n* = 9,473; 4.5%), weight (13,847; 6.5%), BMI (*n* = 19,703; 9.3%), smoking (*n* = 8,801; 4.1%), and Apgar (*n* = 803; 0.4%).

^b^Women with elective cesarean section or without information on emergency/elective cesarean section were excluded (*n* = 21,306; 10.0%).

^c^Defined as third/fourth-degree perineal tear.

^d^Neonatal morbidity was defined as one or more of the following neonatal complications: seizure, pulmonary hypertension, bronchopulmonary dysplasia, hypoxic ischemic encephalopathy, intraventricular haemorrhage grade III or IV, other intracranial haemorrhage, necrotizing enterocolitis, bone fracture, brachial plexus injury, facial palsy, sepsis, and/or cardiac arrest.

Abbreviations: BMI, body mass index; SD, standard deviation

**Fig 2:**
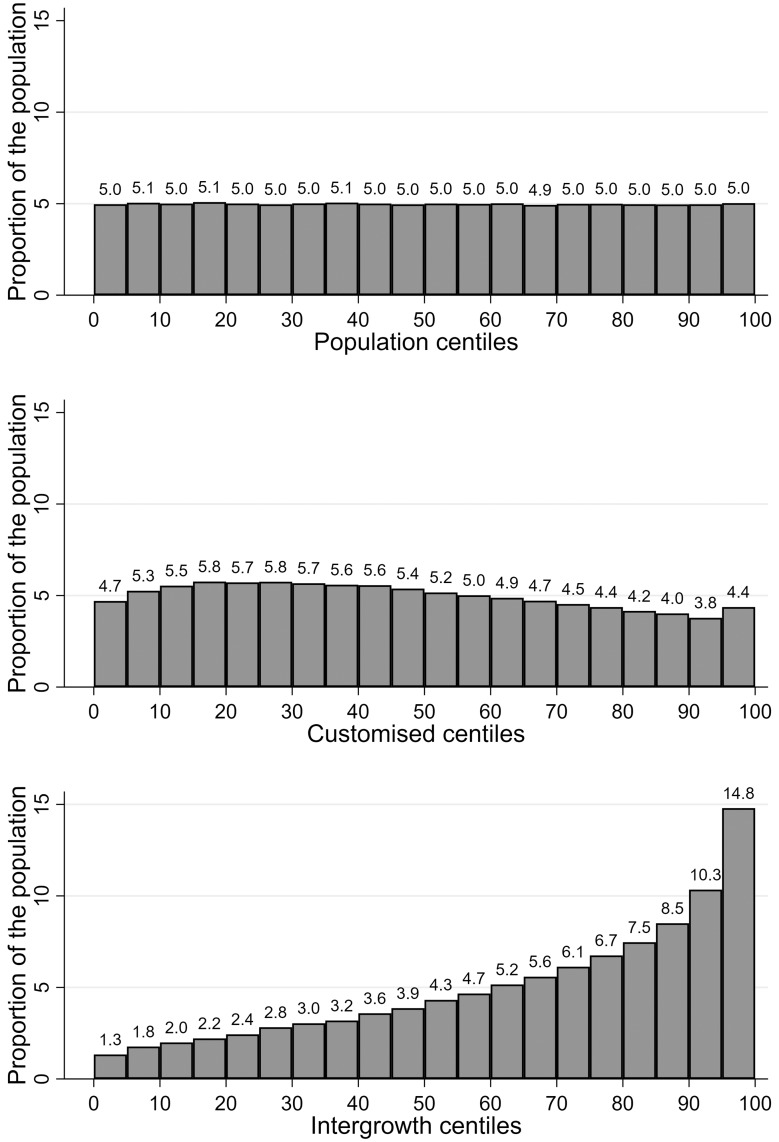
Distribution of Swedish population in groups of 5 centiles^a^ according to different charts. ^a^The proportions for each chart do not add to 100%, because of rounding error.

Customised centiles had an increased proportion of infants below the median (50th centile), whereas assessment using Intergrowth centiles was associated with a marked increased proportion of infants in the upper range (Fig 2). The prevalence of SGA (birth weight < 10th centile) was 10.1%, 10.0%, and 3.1% for population, customised, and Intergrowth centiles, respectively. The prevalence of LGA (birth weight > 90th centile) was 10.0%, 8.2%, and 25.1%, respectively.

**Fig 3:**
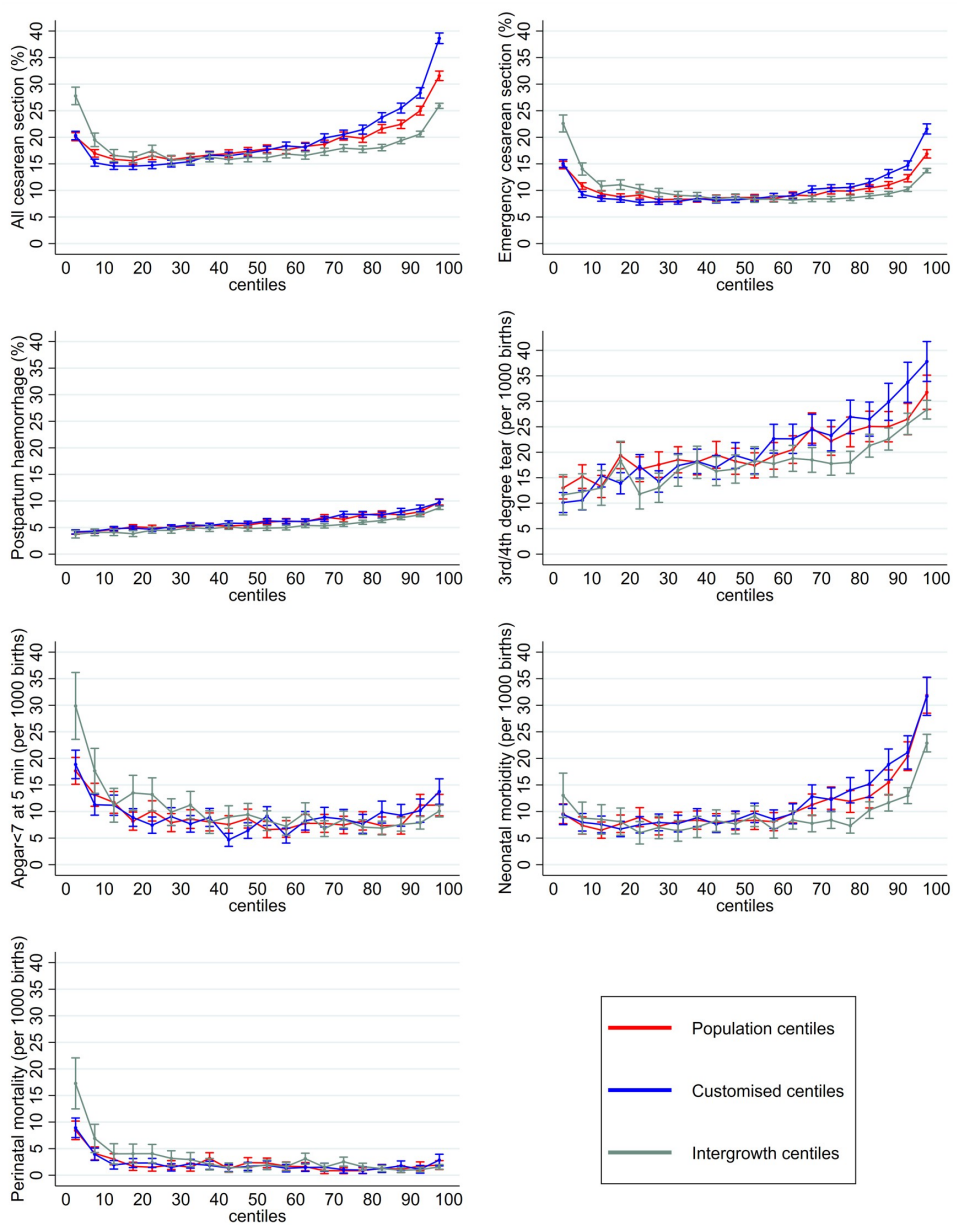
Rate (95% CI) of adverse perinatal outcomes across birth-weight centiles according to population, customised, and Intergrowth charts. CI, confidence interval.

The centile interval that defined the group of infants at lowest risk of adverse outcome differed amongst the maternal and neonatal outcomes studied, across growth charts (Fig 3 and S2 to S9 Tables). The risk of all cesarean section and emergency cesarean section was increased in small and large infants (Fig 3a and 3b) in all charts. The risk for all cesarean section was greater for large infants (‘J’-shaped association), likely related to a higher risk of elective cesarean sections. Birth-weight centiles had a linear association with postpartum haemorrhage and severe perineal trauma, irrespective of the chart (Fig 3c and 3d). Apgar below 7 at 5 minutes and perinatal mortality had an inverted ‘J’-shaped association with birth-weight centiles, reflecting greater risk in small infants (Fig 3e and 3g). A different pattern of association (‘J’-shaped) was observed for the composite of neonatal morbidity, suggesting greater risk in large infants (Fig 3f).

The centile thresholds for population, customised, and Intergrowth charts in which a consistent increase in risk of adverse outcomes was observed are summarised in Table 4, except for postpartum haemorrhage and severe perineal trauma, in which a linear association was observed. The coefficient for the linear association between increasing centiles and greater risk of postpartum haemorrhage was similar across charts (S4 Table). For severe perineal trauma, there was a relevant increase in the coefficient for linear association in relation to customised centiles (S5 Table). The association of birth-weight centiles with perinatal mortality was markedly increased (i.e., doubling of OR) below the 10th centile for population and customised charts and below the 25th centiles for the Intergrowth chart (S9 Table). A full description of the size of effect (OR) across birth-weight centiles for all charts is provided in S2 to S9 Tables.

**Table 4:** Summary of centile thresholds in which a consistent increase in risk of adverse outcomes was observed on population, customised, and Intergrowth centiles.

	Thresholds for risk in small infants			Thresholds for risk in large infants		
Adverse outcome^a^	Population	Customised	Intergrowth	Population	Customised	Intergrowth
All CSs	<5th	<5th	<10th	>60th	>65th	>65th
Emergency CSs	<15th	<10th	<30th	>70th	>65th	>85th
Apgar < 7 at 5 minutes	<15th	<20th	<25th	>90th	>80th	>95th
Neonatal morbidity^b^	-	-	<5th	>65th	>65th	>80th
Perinatal mortality	<15th	<10th	<35th	-	>95th	-

^a^Postpartum haemorrhage and severe perineal trauma were not included in this table, because a linear association was observed (reduced risk in small infants and increased risk in large infants).

^b^Neonatal morbidity was defined as one or more of the following neonatal complications: seizure, pulmonary hypertension, bronchopulmonary dysplasia, hypoxic ischemic encephalopathy, intraventricular haemorrhage grade III or IV, other intracranial haemorrhage, necrotizing enterocolitis, bone fracture, brachial plexus injury, facial palsy, sepsis, and/or cardiac arrest.

Abbreviation: CS, cesarean section

A description of chart-specific thresholds for this study population is provided in Table 2. The strength of association of the chart-specific thresholds for this population with each adverse outcome was broadly similar for all three charts (S10 to S16 Tables). However, increased risk for Apgar < 7 at 5 minutes was observed closer to the reference group for customised centiles compared with other charts (S14 Table). Furthermore, customised centiles remained the only chart to identify a trend of increased risk of perinatal mortality in overgrown infants (above the 95th centile or in the upper 5% of the population; S16 Table).

The performance of charts in the detection of neonatal morbidity and perinatal mortality using thresholds related to fixed FPRs of 10% is described in Table 5. In smaller infants, different thresholds in population (<10.0th centile), customised (<10.1st centile), and Intergrowth charts (<25.5th centile) achieved a similar sensitivity for perinatal mortality (29% for any of the charts). For larger infants, sensitivities were also similar for FPRs of 10% (Table 5). This observation of similar sensitivities between charts for each given fixed FPR was constant for both SGA and LGA infants and across the different outcomes explored, except for a marginal difference for large infants at an FPR of 5% for perinatal mortality in which customised centiles performed slightly better (S20 Table). A full description, including rate, sensitivity, and FPR, for all permutations of thresholds (i.e., fixed centiles thresholds and fixed FPRs) for SGA and LGA relative to neonatal morbidity and perinatal mortality is provided in the supplementary tables (S17 to S20 Tables).

**Table 5:** Diagnostic test performance of different centile thresholds for SGA and LGA at fixed FPRs.

Neonatal outcome related to each chart	Centile threshold	Number of events	Rate (/1,000)	Sensitivity	FPR
**Small infants—FPR10%**					
Neonatal morbidity^a^					
Population	<9.9	179	8.5	8 (7–9)	10 (10–10)
Customised	<10.0	183	8.7	8 (7–9)	10 (10–10)
Intergrowth	<25.4	179	8.5	8 (7–9)	10 (10–10)
Perinatal mortality					
Population	<10.0	133	6.3	29 (25–33)	10 (10–10)
Customised	<10.1	133	6.3	29 (25–33)	10 (10–10)
Intergrowth	<25.5	135	6.3	29 (25–34)	10 (10–10)
**Large infants—FPR10%**					
Neonatal morbidity^a^					
Population	>89.8	554	25.9	24 (22–25)	10 (90–90)
Customised	>87.5	539	25.2	23 (21–25)	10 (90–90)
Intergrowth	>96.9	56	26.2	24 (22–26)	10 (90–90)
Perinatal mortality					
Population	>89.9	39	1.8	9 (6–11)	10 (10–10)
Customised	>87.7	44	2.1	10 (7–13)	10 (10–10)
Intergrowth	>97.0	33	1.6	7 (5–10)	10 (10–10)

^a^Neonatal morbidity was defined as one or more of the following neonatal complications: seizure, pulmonary hypertension, bronchopulmonary dysplasia, hypoxic ischemic encephalopathy, intraventricular haemorrhage grade III or IV, other intracranial haemorrhage, necrotizing enterocolitis, bone fracture, brachial plexus injury, facial palsy, sepsis, and/or cardiac arrest.

Abbreviations: FPR, false-positive rate; LGA, large for gestational age; SGA, small for gestational age

## Discussion

### Main findings

In this Swedish cohort, population, customised, and Intergrowth charts identified a different proportion of SGA and LGA infants, with the greatest difference observed with Intergrowth standard compared with customised standard and population reference. The centile interval reflecting the group of infants at lowest risk for maternal and neonatal outcomes differed by outcome. The threshold associated with increased risk was different according to the chart used for both large and small infants; the strength of association with adverse outcomes (OR) was also different for infants below fixed thresholds (i.e., below the 5th centile) for each chart. However, the strength of association with adverse outcomes for population, customised, and Intergrowth centiles was very similar when assessing chart-specific thresholds for this population (i.e., thresholds that determine 20 equally sized groups with 5% of the population). This suggests the use of different thresholds accounts for a considerable part of the difference between these charts. For fixed FPRs, different thresholds for each chart achieved similar sensitivity for neonatal morbidity and perinatal mortality.

### Interpretation and implication of findings

Despite the association between birth weight above the 90th or below the 10th centiles and adverse perinatal outcomes [20], these are nonetheless thresholds derived from statistical definitions. Consistent with previous findings, our results suggest that thresholds different to the traditional 10th or 90th centiles should be considered [21–24]. A lower risk of neonatal outcomes was observed in large infants in this study, except for neonatal morbidity, which was largely driven by fractures and related morbidity (S1 Table). However, the lowest risk for maternal adverse outcomes was observed in smaller infants. Given that birth-related complications differ by fetal size, our a priori decision to define the group with optimal growth (reference group) as infants in the middle of the distribution (usually between the 40th and the 60th centiles) seems reasonable to achieve a balance between competing maternal and neonatal risk.

Because of the increasing use of interventions for infants identified with fetal growth abnormalities, usually related to induction of labour at early term, caution is needed when selecting the threshold used to define these conditions, as sensitivity and FPRs usually increase in parallel. As an example, recent studies comparing universal third-trimester scan with standard care in the United Kingdom (third-trimester scan performed only if clinically indicated) have indeed shown increased detection of both SGA and LGA, but at a cost of an increase in the FPR, preventing recommendation in clinical practice [25,26]. Similarly, the use of thresholds higher than the 10th and lower than the 90th centiles to define SGA or LGA, respectively, will increase both sensitivity and FPRs. The use of the 25th and the 85th population centiles, proposed by Iliodromiti and colleagues, would identify 40% of the population with abnormal fetal growth potentially requiring early delivery [3]. They have estimated the number needed to treat (NNT) to avoid a perinatal death as 721 (95% CI 598–947). Researchers and clinicians need to agree on the clinically meaningful increase in risk (or the absolute rate) of perinatal mortality that would justify interventions and the level of FPRs (or NNT) that would be acceptable as a cost for an increase in sensitivity. This is an essential step for development of an evidence-based threshold for fetal growth abnormalities that is clinically relevant and not only statistically significant.

Our results can be interpreted in the context of previous literature comparing different charts. Recent large studies have shown that the use of Intergrowth centiles resulted in a reduction in the prevalence of SGA compared with population or customised centiles. These missed SGA infants were at increased risk of stillbirth [12–14]. Intergrowth also identified a considerable increase in the prevalence of LGA, but these infants were at reduced risk of stillbirth [13,14]. Our study not only confirms these previous findings but also adds to previous knowledge by showing that Intergrowth had a comparable association with adverse outcomes when chart-specific thresholds were used. We have observed that the Swedish population sits to the right of Intergrowth birth-weight standards, consistent with the findings from other high-income countries [13,14,27].

With regard to the comparison of population and customised centiles, some differences were observed in this study that are not only related to the shift of birth-weight distributions. The linear association with severe perineal trauma was stronger for customised centiles (i.e., clinically relevant increase in the coefficient), and this was also the only chart to identify increased risk of perinatal mortality in large infants (>95th centile). However, these differences were not reflected by a relevant improvement in the diagnostic test performance in the present study. Our findings suggest that any chart could be used in clinical practice as long as a chart-specific threshold for each population is used to define SGA and LGA. Future studies should explore whether these findings are valid for other populations.

### Study strengths and limitations

The strengths of our study include the use of an established national registry and measured maternal weight. In addition, appropriate methodology was used to deal with the major issues of comparing growth charts. First, we included only term infants to avoid the issues related to differences in birth-weight charts and fetal charts. Second, diagnostic test performance was assessed using fixed FPRs so that the results observed would not merely represent the different trade-offs between sensitivity and specificity related to the use of stricter or broader thresholds. Third, the reference group was restricted to the middle of the distribution because infants closer to the boundaries of 10th and 90th centiles are usually at higher risk.

Some considerations need to be made for appropriate interpretation of our results. We acknowledge this is a homogeneous population in which 80% of women were born in Sweden and prevalence of maternal obesity was low (<10%). Customised centiles may perform differently in a heterogeneous population. The use of ethnicity (if available) instead of country of birth may have provided more appropriate information for customisation. Our sample selection was limited by inclusion of women with data available in the Swedish Pregnancy Registry (first-trimester screen data), which included approximately 20% of all deliveries in Sweden during the study period. In this sample, the proportion of women with age above 35 years was 41% compared with 20% observed in a previous report including all women in a similar period [28]. Other demographic characteristics of this study population and the previous report are described in S21 Table. Finally, these findings might not be translatable to other populations. Differences in population demography as well as obstetric practice between countries may influence this association. Future validation studies in other populations are required to determine the generalisability of our findings.

## Conclusion

Our findings have advanced the debate on the choice of growth charts in clinical practice by acknowledging shifts in birth-weight distribution of the study population in relation to the reference population used to develop each chart, recognising the need for chart-specific thresholds to identify pregnancies at similar risk. This is imperative because ORs and sensitivities are not directly comparable if there is a difference in the FPR. Researchers and clinicians need to agree on what equates to a clinically meaningful increase in the risk of adverse outcomes and an acceptable FPR (or NNT) so that studies can further refine the most appropriate threshold for defining SGA and LGA.

## Supporting information

S1 STROBE Checklist (DOCX)

S1 Text Analysis plan. (DOCX)

S1 Table Prevalence of each neonatal complication. (XLSX)

S2 Table Association of birth-weight charts (fixed intervals of 5 centiles) and all cesarean section. (XLSX)

S3 Table Association of birth-weight charts (fixed intervals of 5 centiles) and emergency cesarean. (XLSX)

S4 Table Association of birth-weight charts (fixed intervals of 5 centiles) and postpartum haemorrhage. (XLSX)

S5 Table Association of birth-weight charts (fixed intervals of 5 centiles) and severe perineal trauma. (XLSX)

S6 Table Association of birth-weight charts (fixed intervals of 5 centiles) and Apgar < 7 at 5 minutes. (XLSX)

S7 Table Association of birth-weight charts (fixed intervals of 5 centiles) and Apgar at 5 minutes (continuous/ordinal outcome). (XLSX)

S8 Table Association of birth-weight charts (fixed intervals of 5 centiles) and neonatal morbidity. (XLSX)

S9 Table Association of birth-weight charts (fixed intervals of 5 centiles) and perinatal mortality. (XLSX)

S10 Table Association of birth-weight charts (fixed intervals of 5% of the population) and all cesarean section. (XLSX)

S11 Table Association of birth-weight charts (fixed intervals of 5% of the population) and emergency cesarean section. (XLSX)

S12 Table Association of birth-weight charts (fixed intervals of 5% of the population) and postpartum haemorrhage. (XLSX)

S13 Table Association of birth-weight charts (fixed intervals of 5% of the population) and severe perineal trauma. (XLSX)

S14 Table Association of birth-weight charts (fixed intervals of 5% of the population) and Apgar < 7 at 5 minutes. (XLSX)

S15 Table Association of birth-weight charts (fixed intervals of 5% of the population) and neonatal morbidity. (XLSX)

S16 Table Association of birth-weight charts (fixed intervals of 5% of the population) and perinatal mortality. (XLSX)

S17 Table Diagnostic test performance of different centile thresholds for SGA in relation to neonatal morbidity. SGA, small for gestational age.
(XLSX)

S18 Table Diagnostic test performance of different centile thresholds for SGA in relation to perinatal mortality. SGA, small for gestational age.
(XLSX)

S19 Table Diagnostic test performance of different centile thresholds for LGA in relation to neonatal morbidity. LGA, large for gestational age.
(XLSX)

S20 Table Diagnostic test performance of different centile thresholds for LGA in relation to perinatal mortality. LGA, large for gestational age.
(XLSX)

S21 Table Demographic characteristics of this study population and the previous report using all SMBR records. SMBR, Swedish Medical Birth Registry.
(XLSX)

## Abbreviations

BMI: body mass index
CI: confidence interval
FPR: false-positive rate
ICD-10: *International Classification of Diseases*, *10th version*
LGA: large for gestational age
NNT: number needed to treat
OR: odds ratio
SD: standard deviation
SGA: small for gestational age
SMBR: Swedish Medical Birth Registry
