# Supplementary material for: Determination of birth-weight centile thresholds associated with adverse perinatal outcomes using population, customised, and Intergrowth charts: A Swedish population-based cohort study
Source: PLoS Med. 2019 Sep 20;16(9):e1002902. doi: 10.1371/journal.pmed.1002902 (PMC6754137; doi:10.1371/journal.pmed.1002902)
Supplement: S1 Text — (DOCX) [file pmed.1002902.s002.docx]

**Determination of birthweight centile thresholds associated with adverse perinatal outcomes using population, customised and Intergrowth charts: A Swedish population-based cohort study**

**ANALYSIS PLAN**

Authors: Matias C Vieira, Sophie Relph, Paul T Seed, Martina Persson, and Dharmintra Pasupathy.

| Objective | To explore different thresholds associated with increased risk of adverse perinatal outcomes using population, customised and intergrowth centile charts. |
| --- | --- |
| Study design and population | Cohort study - linkage of Swedish Registries.  Women with missing data on birthweight and gestational age at birth, preterm or post term birth, multiple pregnancy and fetal abnormalities will be excluded. |
| Outcomes | Maternal: Caesarean section, emergency caesarean section, postpartum haemorrhage, and 3-4^th^ degree tear.  Neonatal: Apgar <7 at 5 minutes, neonatal morbidity and Perinatal Mortality Rate (PMR) |
| Factors or exposures | Birthweight centiles by population charts.  Birthweight centiles by customised charts.  Birthweight centiles by Intergrowth charts. |
| Statistical methods | Data exploration, checking and management.  Application of exclusion criteria.  Assessment of missing data for exposures and outcomes.  Calculation of birthweight centiles: use bulk calculator for customised centiles, use intergrowth calculator for Intergrowth centiles, and calculate population centiles internally developed using this population.  Describe the prevalence of outcomes in relation to birthweight percentiles (grouped in interval of 5 units of percentile) and in relation to groups of 5% of the population ranked by birthweight centiles.  Logistic regression to assess risk of outcomes across birthweight centiles for each definition.  Assess diagnostic test performance of each chart in predicting adverse neonatal outcomes. To consider different permutation of false positive rates.  Post hoc (suggested by reviewer): Sensitivity analysis using ordinal logistic regression to explore Apgar as continuous |
